# Origami-inspired thin-film shape memory alloy devices

**DOI:** 10.1038/s41598-021-90217-3

**Published:** 2021-05-26

**Authors:** Prasanth Velvaluri, Arun Soor, Paul Plucinsky, Rodrigo Lima de Miranda, Richard D. James, Eckhard Quandt

**Affiliations:** 1 Chair for Inorganic Functional Materials, Faculty of Engineering, Kiel University, Kiel, Germany; 2 Mathematics Institute, University of Oxford, Oxford, UK; 3 Aerospace and Mechanical Engineering, University of Southern California, Los Angeles, USA; 4 Acquandas GmbH, Kiel, Germany; 5 Aerospace and Mechanical Engineering, University of Minnesota, Minneapolis, USA

**Keywords:** Biomedical engineering, Implants, Design, synthesis and processing, Surface patterning, Applied mathematics

## Abstract

We describe the design and fabrication of miniaturized origami structures based on thin-film shape memory alloys. These devices are attractive for medical implants, as they overcome the opposing requirements of crimping the implant for insertion into an artery while keeping sensitive parts of the implant nearly stress-free. The designs are based on a group theory approach in which compatibility at a few creases implies the foldability of the whole structure. Importantly, this approach is versatile and thus provides a pathway for patient-specific treatment of brain aneurysms of differing shapes and sizes. The wafer-based monolithic fabrication method demonstrated here, which comprises thin-film deposition, lithography, and etching using sacrificial layers, is a prerequisite for any integrated self-folding mechanism or sensors and will revolutionize the availability of miniaturized implants, allowing for new and safer medical treatments.

## Introduction

Origami, the ancient art of paper folding, describes rules for designing and folding a pattern idealized as an assembly of rigid panels and flexible hinges^1^. The folding pattern for origami is typically fabricated from a flat 2D sheet, then deployed to a complex 3D shape by folding. Each pattern is subject to strong geometric constraints, directly linking the pattern design and desired final shape. These constraints also facilitate a robust and controlled deployment path for the pattern, even during large deformations. As a result, functional devices, which use origami structures as a template, are being explored in a myriad of engineering domains. Examples include mechanical and optical metamaterials^2,3^, stretchable and conformable electronics^4^, biosensors^5^, deployable space structures^6^, and large-amplitude actuators^4^.

An important potential application of miniaturized origami structures is implant design. Implants are a central instrument of modern medical treatments, as they are an essential component to minimally invasive procedures. A key example is the treatment of brain aneurysms. The implant should be both capable of being crimped and inserted into a small catheter and capable of expansion upon exiting the catheter. In this setting, shape memory alloys are of great interest because their superelasticity enables superior crimpability, and their shape-memory enables self-expansion when exiting the catheter^7^ at body temperature. As a result, marrying origami-based implant structures with thin-film shape memory technology is attractive: (1) It enables a fundamental reduction in the implants' size through crimping by folding. (2) Origami-folding also offers large regions (panels) designed to be stress-free during folding and unfolding. Thus, through proper design and fabrication, origami panels may be suitable as support structures for sensitive components. Although they are theoretically modeled as stress-free, the experimental case is not ideal and can result in specific stresses usually in the vicinity of the hinges. This feature could facilitate promising technologies, including drug reservoirs and components for future intelligent implants such as sensors or antennas for energy and data transmission^8^.

A main challenge associated with origami-based implant design is that at least two stable configurations are needed, one with maximum possible folding (to place into the catheter) and the second one, consisting of a stable 3D shape upon unfolding (deployed). Typically, changing between these two states requires the application of forces, which is often realized through balloon dilatation in the case of implants. This procedure is considered to be delicate and challenging. An attractive alternative is to use shape memory materials in the implant design because they undergo a phase transformation where the high-temperature phase (austenite) will restore the original (unfolded) shape simply by natural heating to body temperature^9^. Previous studies report a shape memory stent graft using a waterbomb origami pattern (unit cell area of 100 mm^2^ being much too large for typical medical implant requirements in the brain)^10^, fulfilling the above criteria of having two stable states.

The fabrication route for implant design needs to provide the possibility for miniaturization, cost-effective fabrication, and a short processing time, the latter being a prerequisite for any patient-specific implants. Furthermore, it should enable the monolithic integration of future concepts related to self-folding mechanisms and components needed for intelligent implants. A promising approach that overcomes the limitations of classical fabrication methods, such as laser cutting of shape memory tubes^11^ or woven structures using shape memory wires^12^, is a wafer-based monolithic process comprised of thin-film deposition, lithography, and etching using sacrificial layers^13^ that precisely define the folds.

In this paper, we demonstrate this origami-adapted thin-film fabrication method, and we introduce a systematic and flexible approach to origami implant design based on a group theory method. In the group theory method, we begin with a description of a group of transformations, such as a helical or conformal group, that preserve the rigidity of individual tiles. All the design is then done at the level of the unit cell. The main result is that, if the elements of the group commute (i.e., it is an Abelian group), and the unit cell is made compatible with its nearest neighbors at the boundary hinges, then application of the full group to the unit cell automatically builds a fully compatible, whole structure^14–16^. In this way, the parameters governing the geometry and foldability of the full structure are encoded in the unit cell design parameters and the group parameters; this allows for a simple and general encoding of the geometry that is desirable for patient-specific designs. The group theory method also produces structures that can exhibit constructive/destructive interference with suitable radiation^17^, analogous to phased arrays but non-periodic, that opens the way towards remote and noninvasive diagnostics of the structure. The thin-film shape memory technology is demonstrated on one such design, the so-called waterbomb tube, with the fabrication of multiple devices of varying porosities. The devices have a unit cell size of 9 mm^2^ and achieve stent-like shapes when unfolded. The films are manually folded to achieve the devices, consistent with the derived folding kinematics, and characterized for their expansion in a simulated vessel on heating.

Our long-term ambition is to develop a versatile platform for the design and fabrication of complex and functional miniaturized implants and devices—one that fully integrates advances in thin-film shape memory technology with origami design principles. The design, fabrication, and deployment of a waterbomb stent prototype demonstrated here serves as a proof-of-concept in this direction.

## Results and discussion

### Theoretical: the group theory approach for stent design

**Figure 1:**
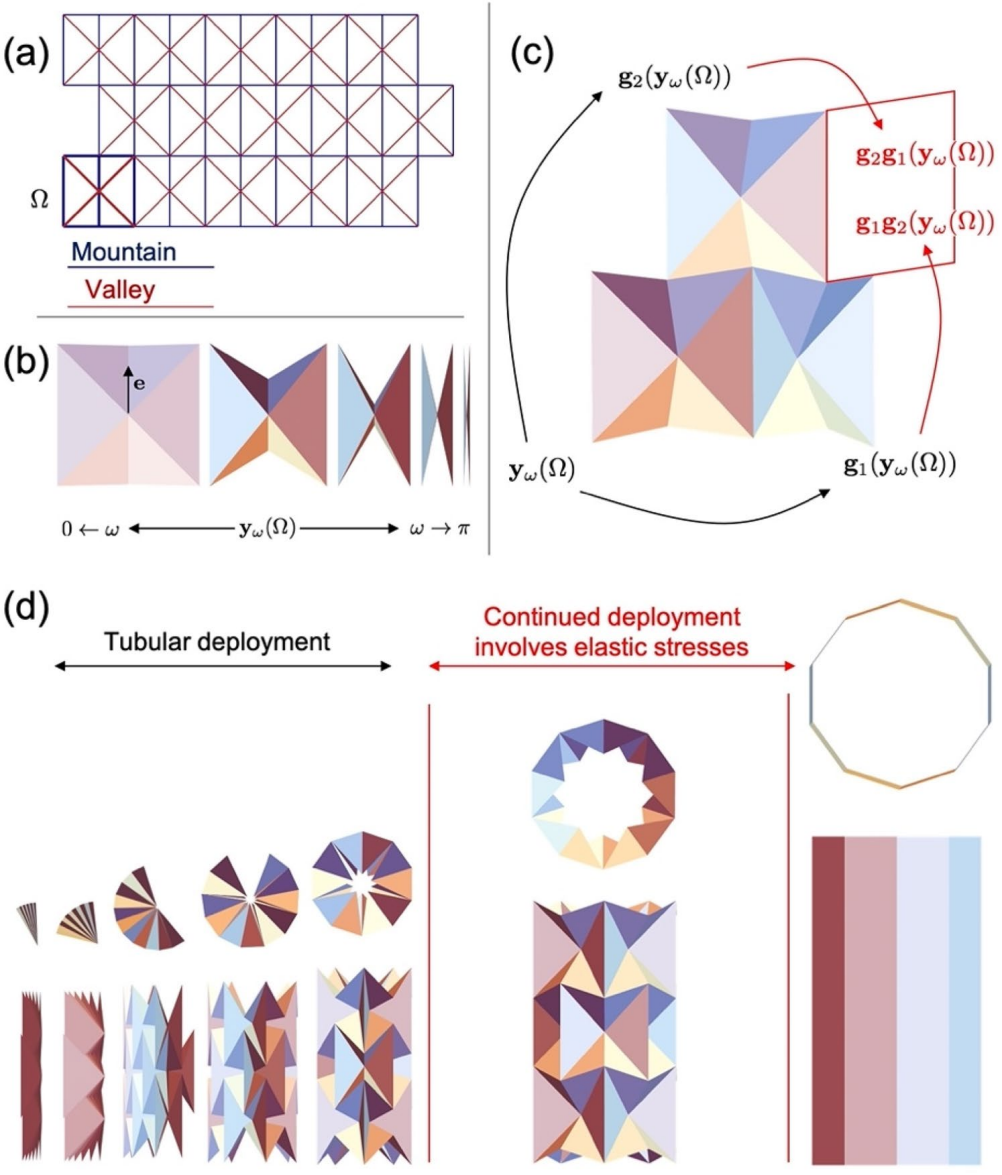
Group theory approach to waterbomb origami. (**a**) The crease pattern, unit cell, and mountain-valley assignment associated with this origami. (**b**) Symmetric kinematics of the unit cell. The axis of symmetry is indicated by ${\mathbf{e}}$. (**c**) Method to obtain an overall origami structure by application of isometries to the unit cell. (**d**) The origami structures involve unfolding from a compact crimped state into potentially three perfect tubes of expanding radius. After unrolling to the first compact tube, the structure can no longer unfold by an ideal origami motion to achieve the other tubular states. The image is made using Mathematica (V 12, https://www.wolfram.com/mathematica/).

The group theory approach to origami is a recipe for designing and folding structures made of repeated unit cells. To fix the ideas, we introduce our approach in the context of the well-known waterbomb origami^18^. The flat crease pattern of this origami (Fig. 1a) can be obtained by repeating the square unit cell ${\Omega }$ shown in bold. Let us first imagine cutting-out and folding this cell in isolation (Fig. 1b). As the figure highlights, it is possible to show that the cell is "rigidly foldable," i.e., without stretch or flexure in the six panels, by a one-parameter family of deformations. The deformation maintains symmetry across the axis indicated by ${\mathbf{e}}$ and is consistent with the mountain-valley assignment shown. For reference, we label a single folded cell ${\mathbf{y}}_{{\upomega }} \left( {\Omega } \right)$ for a folding angle $\omega$ that parameterizes the fold along one of the (four equivalent) diagonal creases. Per convention in the origami literature, folding angles are $\pi -$ "the dihedral angles" of the folded creases. So $\omega = 0$ gives the flat cell, $\omega = \pi$ the completely folded cell, and $0 < \omega < \pi$ a cell that has been partially folded.

We can repeat the unit cell by copying it and applying an "isometry" that rotates and translates the copy in 3D space. The copied cell then has the form ${\mathbf{g}}\left( {{\mathbf{y}}_{{\upomega }} \left( {\Omega } \right)} \right)$, where ${\mathbf{g}}\left( {\mathbf{x}} \right) = {\mathbf{Rx}} + {\mathbf{b}}$ defines an isometry with rotation ${\mathbf{R}}$ and translation ${\mathbf{b}}$. In repeating cells in this fashion, the recipe for obtaining an overall origami structure is simple: Identify two such isometries

1 $${\mathbf{g}}_{i} \left( {\mathbf{x}} \right) = {\mathbf{R}}_{i} {\mathbf{x}} + {\mathbf{b}}_{i} ,\;\;\; i = 1,2,$$

that, together with the partly folded unit cell, satisfy the three rules

2 $$\left\{ {\begin{array}{*{20}c} {{\mathbf{g}}_{1} \left( {{\mathbf{y}}_{\omega } \left( \Omega \right)} \right) \;takes \;the\, unit\;cell \;{\mathbf{y}}_{\omega } \left( \Omega \right) \;to\; its \;neighbor\; to\; the \;right;} \\ {{\mathbf{g}}_{2} \left( {{\mathbf{y}}_{\omega } \left( \Omega \right)} \right) \;takes \;the \;unit\; cell \;{\mathbf{y}}_{\omega } \left( \Omega \right) \;to\;its\; neighbor \;above;} \\ {{\mathbf{g}}_{1} \left( {{\mathbf{g}}_{2} \left( {\mathbf{x}} \right)} \right) = {\mathbf{g}}_{2} \left( {{\mathbf{g}}_{1} \left( {\mathbf{x}} \right)} \right) \;for\, all \;x\; in \;{\mathbf{y}}_{\omega } \left( \Omega \right)} \\ \end{array} } \right.$$

The general idea is sketched in Fig. 1c. The first two rules say that the cell and its two transformed copies fit together without gaps. The third, and perhaps the least obvious rule, guarantees that no matter what order the transformations are applied, the same fourth cell results. Once these rules are verified, a whole origami structure is built by repeated applications of ${\mathbf{g}}_{1}$ and ${\mathbf{g}}_{2}$ to the unit cell.

The recipe expressed with Eqs. (1–2) is fundamentally related to group theory. The collection of all products of the two isometries above,

3 $${\mathcal{G}} = \left\{ {{\mathbf{g}}_{1}^{p} {\mathbf{g}}_{2}^{q} :p,q \in \left\{ { \ldots , - 2, - 1,0,1,2, \ldots } \right\}} \right\},$$

is a group of isometries. These groups catalog symmetry and provide a precise way to organize unit cells—indexed by integers $p, q$—on an overall structure. As an example, the crease pattern in Fig. 1a is fully described by a translation group with the cell in the top-right corner given by ${\mathbf{g}}_{1}^{3} {\mathbf{g}}_{2}^{2} \left( {\Omega } \right)$ for a suitable choice of isometries in Eq. (1). However, unlike the crystallographic groups for atoms or molecules, symmetry cannot be applied arbitrarily to origami because unit cells must fit together without gaps. The rules in Eq. (2) are the fitting conditions for when the group in Eq. (3) (and appropriate subsets) can be applied to the cell ${\mathbf{y}}_{{\upomega }} \left( {\Omega } \right)$ to produce the full origami structure. An additional condition assures that the group is discrete. Discreteness of the group has the physical meaning that, upon repeated application of the group elements in ${\mathcal{G}}$, we arrive at the seam, i.e., the place where the folded unit cells on the far left of Fig. 1a meet those on the far right of 1a. Discreteness of ${\mathcal{G}}$ assures that these cells also fit together perfectly: there is no identifiable seam.

In the supplement, we explicitly characterize these rules to describe the waterbomb origami that emerges from the flat crease pattern in Fig. 1a using the folded cells in 1d. We note that other approaches characterize symmetric waterbomb origami; notably, the approach is based on spherical linkages^19^. However, the group theory approach is distinctive in its versatility: It is a rare design and characterization method in origami that is not beholden to the topology of the crease pattern. The same general rules in Eqs. (1–3) apply whether we are dealing with waterbomb origami or any other origami with repeated unit cells^14,15^.

The characterization of waterbomb origami, shown graphically in Fig. 1d, has several important features. First, the pattern can be crimped into a fully folded state suited for small-volume packaging, and it naturally deploys by unfolding and rolling up as a tubular structure. After a certain unfolding, it then achieves the first of three "stent-like" perfectly tubular states. By unfolding further, two additional stent-like states with a wider radius are possible, but there is a clear barrier to such transitions. Since the left and right boundary of the structure comes in contact when reaching the compact tube, continued motion becomes limited. As described in the appendix, the structure cannot unfold further by ideal origami deformations since the contact prevents this motion. As a result, the structure undergoes elastic deformation and stresses when transitioning from the compact tube to the fully deployed tube.

## Experimental

### Geometry

**Figure 2:**
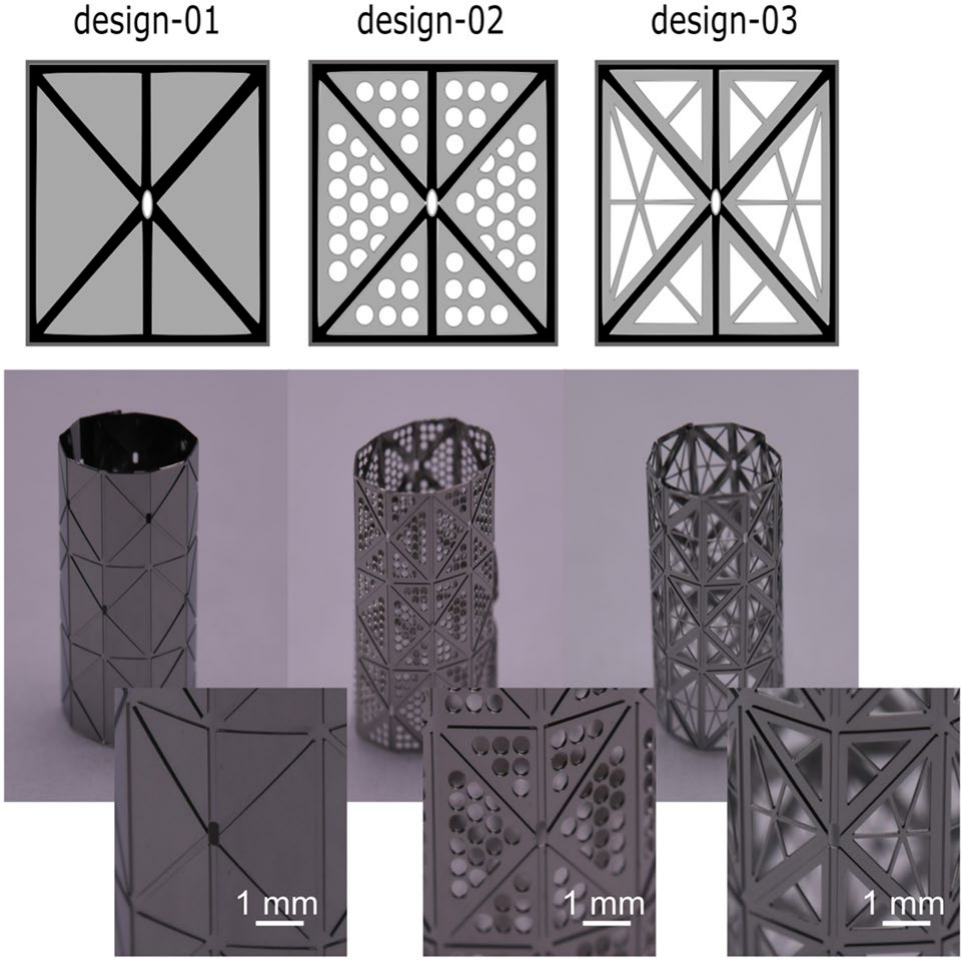
On the top, unit cell designs 01–03 are shown with varying porosities (Table S1), the hinges (black) are 5 µm thick, and the rigid regions (grey) are 40 µm thick. On the bottom, a profile photo showing the fabricated stents with four different geometries with a unit cell length of 3 mm. The inset shows the magnified view of each unit cell. The image is made using Inkscape (V 0.92, https://inkscape.org/).

Thin-film SMAs are fabricated according to the waterbomb design Fig. 1a with a unit cell area of 3 × 3 mm^2^. The film is five unit cells across (15 mm) and three-unit cells long (9 mm) and can take the shape of a stent-like device up to 4.8 mm in diameter. The unit cell is composed of three regions: thick stiff regions that undergo minimal deformation (grey), thin hinged regions that undergo significant deformation on bending (black), and porous regions (white) (Fig. 2, top). The unit cell can be folded along the hinges with four valley folds along the diagonal and two vertical mountain folds by this design. Note that the mountain and valley folds come together at the unit cell's interior vertex under this folding, producing undesirable stress concentration. Therefore, a hole is placed at the center of the unit cell to avoid this feature.

Medical implants are placed in a diseased artery to fulfill a specific task: to keep the artery open in case of strokes or to reduce flow in aneurysm treatment. Nevertheless, they also need a specific porosity to maintain blood flow into side branches. Previously, such porosity was not considered in the implant design, which limits their possible application^10^. However, our thin-film fabrication technology has a high design freedom, which permits the patterning of panels with varying porosities. We demonstrated this by fabricating devices with porosity of 0.5%, 23%, and 33% for designs 1–3 (Fig. 2 and Table S1), respectively. We also demonstrate that the pattern within a panel can be modified for desired porosity without significantly affecting the overall folding process.

### Materials

The shape memory alloys used in the medical industry are austenitic at room temperature (RT) and use the stress-induced martensitic transformation (superelasticity). In a real application, folding would be performed in the martensitic state at cryogenic temperatures. Then, upon placement into the catheter, heating to body temperature results in shape-recovery to the original (desired) unfolded state. For simplicity in the experimental setup, it is beneficial to have shape memory material be martensite at RT to avoid the need to cool to during the folding (crimping). Therefore, the composition of the shape memory alloys is adjusted to obtain martensite at RT. Accordingly, to regain the original shape, one needs to heat into austenite well beyond RT. This shift in the transformation temperature, while a departure from the intended medical implant design, is not expected to have a dramatic effect on the characteristic deployment of the device. Of note, the alloy composition can generally be tuned to lower the transformation temperature without significantly altering shape recovery^9^, and this tuning is certainly possible in the thin-film structures described here^20^. Nevertheless, the testing of shape memory implants at cryogenic temperatures is key to their efficacy in clinical settings and will need to be pursued in the future should the fabrication, and design methods mature to the point of viability.

### Fabrication

The basic routine to fabricate free-standing thin-film structures has been discussed in detail elsewhere^13^. It combines different microsystem technology processes such as lithography, magnetron sputtering, and wet-chemical etching. To obtain more complicated 2.5 D geometries, some of the processes mentioned above have to be repeated or modified^20^. Such 2.5 D structures are essential for the fabrication of the origami structures. It allows for varying the structures' stiffness by varying the thickness of the thick-stiff panels (40 μm) and the thin-flexible hinges (5 μm), as sketched at the top in Fig. 2. As we demonstrate, this freedom of process parameters available using our thin-film technology allows the fabrication of crease patterns essential for an origami structure. The fabricated structures are amorphous, so a heat treatment (550 °C) is done to shape-set (into cylinder of 4.8 mm diameter) and crystallize the films using a rapid thermal annealing apparatus. Films fabricated with different porosities are shown at the bottom of Fig. 2; the inset shows a close-up of a unit cell.

**Figure 3:**
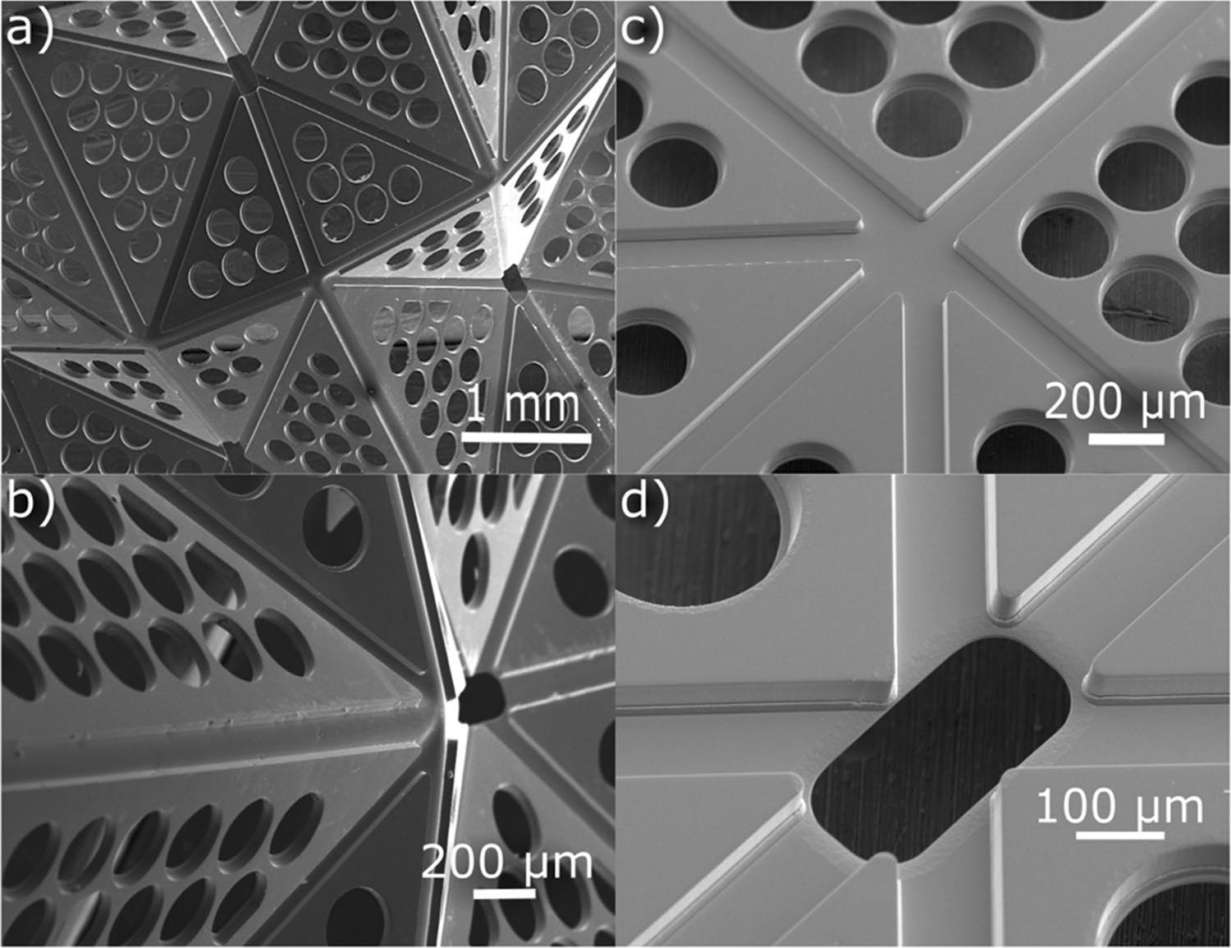
SEM images of the partially folded unit cells of design-02 (**a**) shows the crease pattern for folding (**b**) shows a close-up along the folding line between the unit cells; note the thin and thick regions of the unit cells. Close-up view along the corner (**c**), center (**d**) of the unit cell when unfolded. The image is made using Inkscape (V 0.92, https://inkscape.org/).

The SEM images (Fig. 3a) of the partially folded stent structures show the fine edge quality using thin-film technology. The close-up views (Fig. 3b,c) show the raised thicker regions, which undergo minimal deformation during folding. Most of the folding takes place along the hinges, with the overall deformation expected from the waterbomb design. The center of the unit cell (Fig. 3d), as discussed in methods, is void of material to allow the surrounding mountain-and-valley folding to be free of stress concentrations.

### Deployment

**Figure 4:**
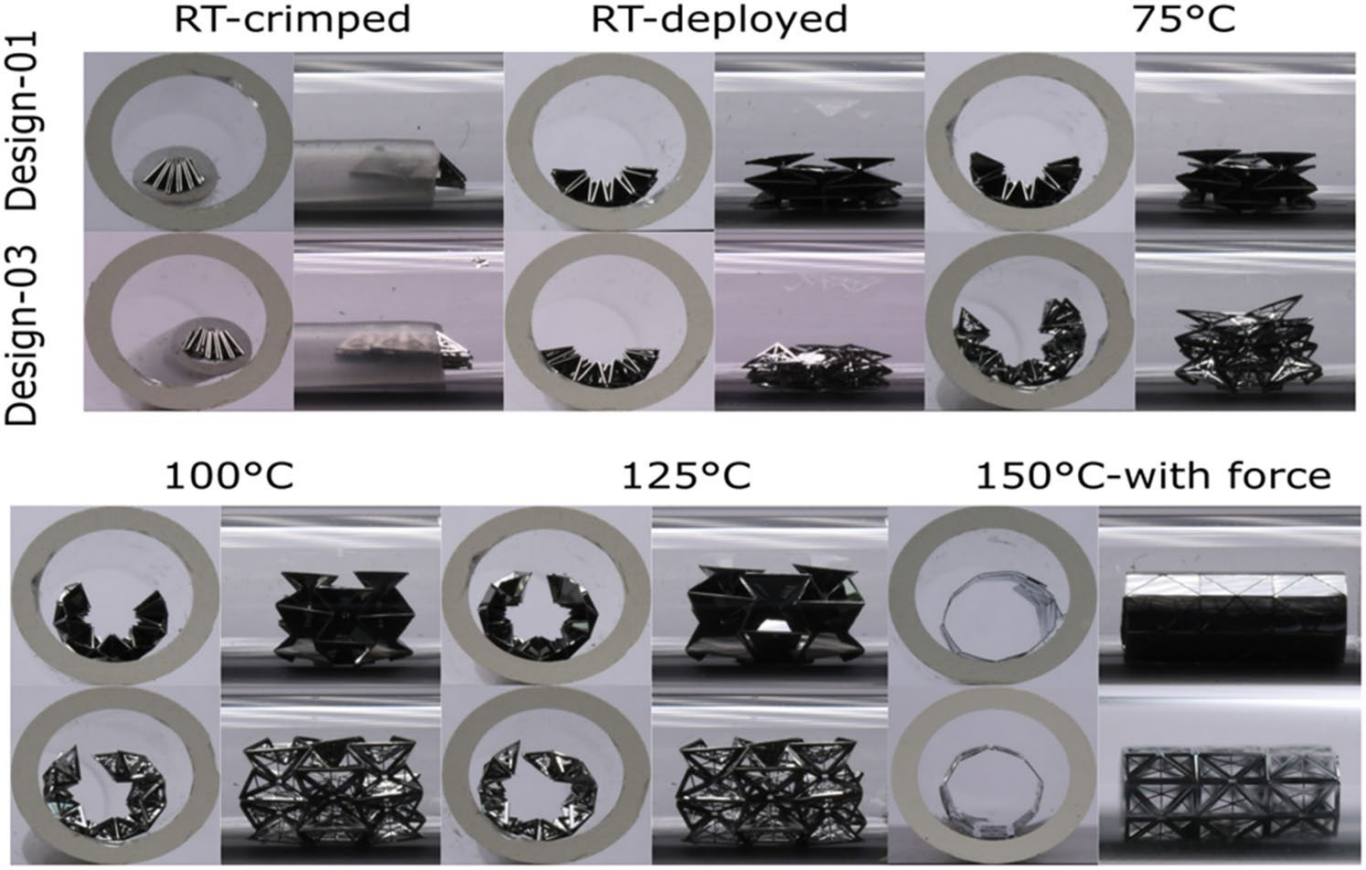
Profile pictures of the stent-devices expanded in a glass tube (Ø 6.3 mm) on heating. Two images (cross-sectional and front view) for each temperature are acquired for designs 1 & 3. The room temperature (RT) images show that the devices are crimped in a catheter of an internal diameter of 2 mm. On heating to 150 °C, the devices did not recover their shape entirely, and a force from the inside was required for complete shape regain. The image is made using Inkscape (V 0.92, https://inkscape.org/).

The stents are folded manually and loaded inside a catheter of 2 mm in diameter that is, in turn, placed inside a simulated vessel (glass tube of Ø 6.3 mm) (Fig. 4, top left). A significant amount of elastic strain in martensite is recovered when the stents exit the catheter at room temperature and are free in the simulated vessel. The series of images (cross-sectional and top view) show the stents gradually transform from martensite into austenite on heating, resulting in deployment towards their original shapes. The austenitic transformation temperature was around 105 °C, so the setup was heated until 125 °C for homogenization. At 125 °C, the complete shape of the stent was not recovered. Further heating to 150 °C did not induce significant additional recovery. On observation, it is seen that the overall stent is in a specific metastable state. One needs to apply a small amount of force from inside, preferably at the center of the unit cells, to regain the complete shape (Fig. 4, bottom right).

The force was quantified to expand the Design-01 stent using a clinical balloon catheter (Invatec, diameter of 5 mm). A pressure of 2 bar was used to inflate the balloon catheter and expand the stent. For perspective, current clinical brain interventions use a pressure of 7 bar for some typical treatments. One can also get a sense of how small this load is by a simple mechanical thought experiment: Suppose the tube were not an origami but instead a homogenous thin-walled cylinder. Then, due to force balance, an internal pressure $p$ as above would generate a hoop stress $\sigma \sim pR/h$ depending on the radius $R$ and thickness $h$ of the thin-walled cylinder. The hoop strain is then estimated as $\varepsilon \sim \sigma /E$ for a characteristic modulus $E$ of the austenite phase. Taking $E = 60 GPa, h = 40 \mu m,$ and $R = 2.4 mm$ to be consistent with our stent design, we obtain the estimate $\varepsilon \sim 0.02 \%$. A hoop strain of this magnitude would produce essentially no discernable deformation in the tube. Since this is obviously not what is happening in our stent, we can reasonably conclude that the large deformation induced by this 2 bar pressure is the result of unfolding along the origami creases.

**Figure 5:**
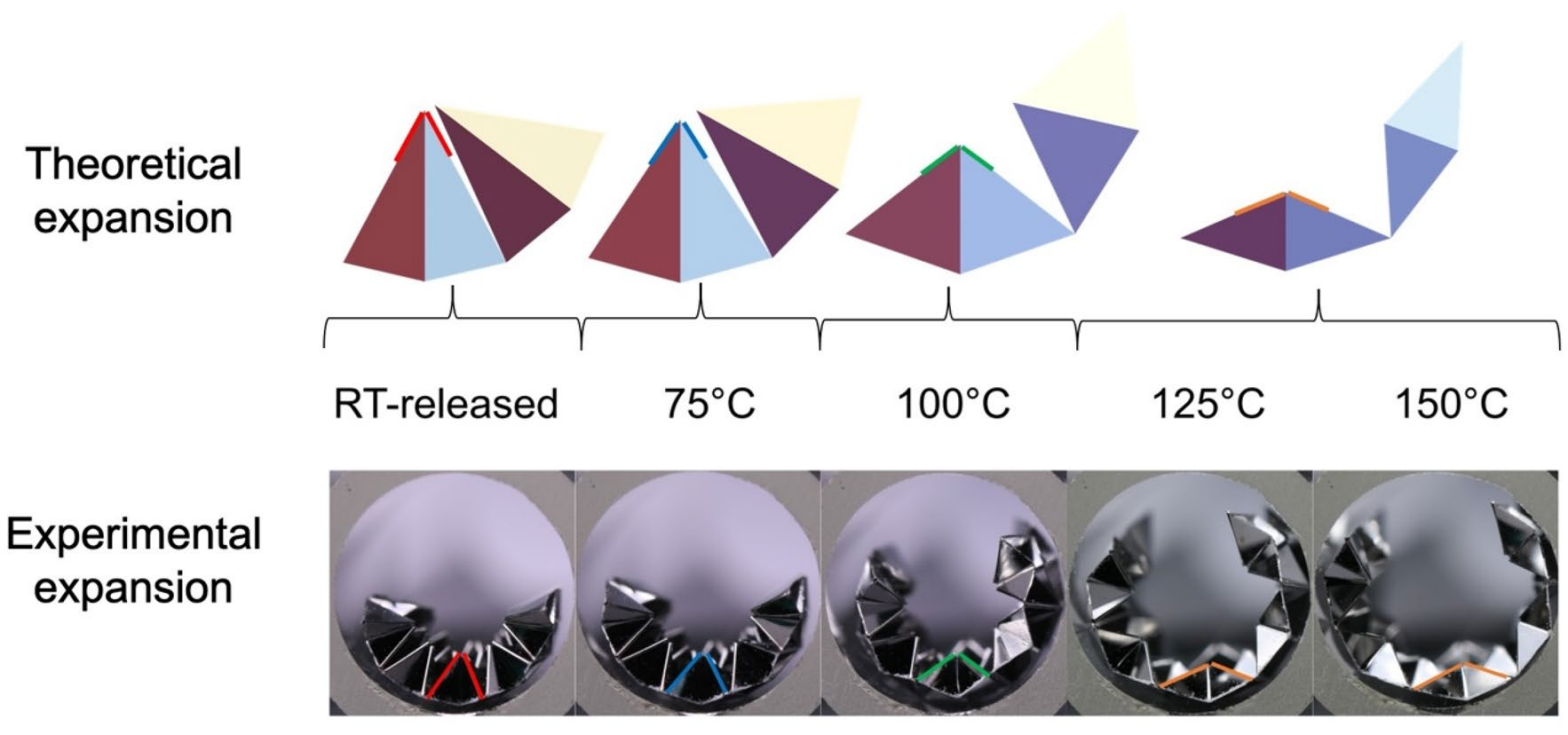
Expansion of devices on heating when expanded on heating theory vs. experimental. For the theory, only two cells within in overall tube are displayed. The theory cells are chosen, as indicated, to match the representative angles in the cells of the experiment. The image is made using Inkscape (V 0.92, https://inkscape.org/) and Mathematica (V 12, https://www.wolfram.com/mathematica/).

The expansion paths observed on heating the devices in the experiments do not follow the exact unfolding path laid out by the theory; instead, the devices expand as arcs of a larger radius. However, at 150 °C, the expansions converge to configurations in line with the theory, though complete unfolding is not obtained due to the aforementioned metastability. The differences between the theory and experiment could be the result of various factors: First, there is no material thickness considered in theory; however, it has a value of 40 μm in the experiment. Second, the plastic deformation and hysteresis also affect how it unfolds on expansion. Third, the theory requires that the unfolding occur in a precise nonuniform way. Specifically, to avoid significant stress in the panels, it is necessary that the folds within the unit cells unfold much faster than the folds between the unit cells. In contrast, during the experiment, the martensitic transformation drives the unfolding to be more uniform during the temperature change. The resulting competition appears to have a strong influence on the expansion in the experiment (Fig. 5). The theory cells are chosen to match the angles of a representative cell in the experiments. One can see that the “V”-shaped angle connecting the neighboring cells is less folded in the experiments. Note this difference is most pronounced at the early stages of deployment, when the differences between folding angles in theory are quite disparate. At the latter stages of deployment (125–150 °C), the comparison between theory and experiment agree.

Regarding the metastable state, in addition to the barriers to deployment discussed previously, local distortions of the hinges during the crimping likely play a role in suppressing full shape recovery. Each hinge has a width of $w \approx$ 100 μm and a thickness of $h = 5$ μm, and thus can be idealized as an Euler–Bernoulli beam in-plane strain during the folding process. At the most crimped state, where the folding angles are given by $\omega = \pi$, the radius of curvature of each hinge is $\rho \sim w/\omega \approx 30$ μm. This calculation suggests that certain creases in the crimped state see maximum tensile and compressive strains on the order of $\varepsilon \sim 8 \%$ (perhaps slightly higher in tension due to the natural tension–compression asymmetry of shape memory alloys). This level of strain can induce plasticity in the hinges that impede full recovery. In addition, the martensite phase, where the crimping is done, is a self-accommodated mixture of twinning microstructures prior to deformation—one that is not biased to prefer tension or compression in any particular direction. Thus, the significant strains at crimping likely induce a complex rearrangement of the martensitic microstructure, which again can be an impediment to full recovery.

On the latter point, the picture can be quite different when one considers superelasticity rather than shape memory. In the superelastic setting, twinning mechanisms are directly induced by stress; they are not present from the onset. As such, there is no need for complex rearrangement of microstructure. This means, at least heuristically, that the path to full shape recovery is more direct: twining arises as a consequence of stress and likely recedes as a consequence of stress relaxation. We have fabricated a device that incorporates aspects of both superelasticity and shape memory and explored its deployment characteristics in a Supplementary Video 1. The device has a low austenite finish temperature of 60 °C and is not purely martensite are RT. As can be seen from the video: Crimping the sample induces a superelastic response. Releasing the load leads to a quick but partial recovery. And heating the sample leads to full recovery. We note that the device is stiff, and shape recovery is abrupt rather than graceful. Nevertheless, these results, when combined with the results based on pure shape memory, suggest a “sweet-spot” in compositional tuning, one where we overcome any potential metastability through folding induced superelasticity but still retain a pattern that is compliant and foldable.

Waterbomb origami has a remarkable range of foldability, from the folded crimped state to the expanded tube. This feature leads to challenges in deployment but is also a key requirement for biomedical applications^10,21^. Future efforts will be directed towards tailoring the unit cell and material composition to suppress deployment barriers without suppressing the range of foldability. For tailoring the origami design, the group theory method^14–16^ has a demonstrable versatility: As discussed, all we need to do is change a single unit cell ${\Omega }$ and parameterize its kinematics. Once done, we can characterize the origami structures emerging from the group approach in Eqs. (1–3) by a process that is no more or less difficult than the characterization on display here for waterbomb origami. Second, one can adapt the alloy composition using localized heat treatments as the transformation of the shape memory alloys (NiTi system) strongly depends on Ni composition^9^. A localized heat treatment (using laser beams^22^) could change the transformation temperatures between the panels and hinges (and even different hinges), thereby allowing us to introduce superelastic effects as needed and to control the sequence of unfolding. These features could help to accommodate the natural folding motion of the origami device and overcome barriers to full shape recovery. Even so, there are other challenges: The waterbomb devices here, for instance, have high stiffnesses that only allow modest curvature in bending. This fact limits their use in neurological applications, where the implants have to be extremely flexible to be deployed in the highly tortuous brain vessels. Thus, a further ambition is to explore a more flexible design with similar foldability.

## Conclusion

To summarize, our study is the first to realize millimeter-scale origami using thin-film shape-memory materials with the feasibility to miniaturize into microscale. This work can be adapted in the future with the help of group theory and other novel approaches in origami design that have demonstrated, through optimization methods, crease patterns that obtain complicated 3D shapes on folding^23,24^. Notably, a variety of curved shapes can often be found by optimizing slight perturbations of patterns that emerge from a group theory approach. Such patterns can be vital in designing patient-specific implants. The only limiting factor appears to be the ability to fold these micro-scale structures and achieve desirable stiffness/flexibility in the structure. Our thin-film shape memory technology can provide a pathway to overcome these limitations and revolutionize the availability of miniaturized implants for safer medical treatments.

## Methods

### Kinematics of the unit cell

The parametrization of the unit cell is derived generically as follows. Let ${\mathbf{e}}_{1} ,{\mathbf{e}}_{2} ,{\mathbf{e}}_{3}$ denote the standard basis vectors on ${\mathbb{R}}^{3}$ and fix unit tangent vectors ${\mathbf{t}}_{1} , \ldots ,{\mathbf{t}}_{6}$ perpendicular to ${\mathbf{e}}_{3}$. These tangents parametrize the crease pattern, which we wish to deform according to a rigid folding motion about the central vertex, assumed to be located at the origin $0$. This means that the deformed tangents, which we denote by ${\mathbf{t}}_{1}^{^{\prime}} , \ldots ,{\mathbf{t}}_{6}^{^{\prime}}$, are obtained by successive rotations about the preceding crease lines^25^:

$$\begin{gathered} \begin{array}{ll} {{\mathbf{t^{\prime}}}_{1} = {\mathbf{t}}_{1} } \\ {{\mathbf{t^{\prime}}}_{2} = {\mathbf{R}}_{{{\mathbf{t}}_{1} }} \left( {{\upgamma }_{1} } \right){\mathbf{t}}_{2} ,} \\ {{\mathbf{t^{\prime}}}_{2} = {\mathbf{R}}_{{{\mathbf{t}}_{1} }} \left( {{\upgamma }_{1} } \right){\mathbf{t}}_{2} ,} \\ {{\mathbf{t^{\prime}}}_{3} = {\mathbf{R}}_{{{\mathbf{t}}_{1} }} \left( {{\upgamma }_{1} } \right){\mathbf{R}}_{{{\mathbf{t}}_{2} }} \left( {{\upgamma }_{2} } \right){\mathbf{t}}_{3} ,} \\ \vdots \\ \end{array} \hfill \\ {\mathbf{t}}_{1}^{^{\prime}} = {\mathbf{R}}_{{{\mathbf{t}}_{1} }} \left( {{\upgamma }_{1} } \right) \cdots {\mathbf{R}}_{{{\mathbf{t}}_{6} }} \left( {{\upgamma }_{6} } \right){\mathbf{t}}_{1} , \hfill \\ \end{gathered}$$

where ${\mathbf{R}}_{{{\mathbf{t}}_{1} }} \left( {{\upgamma }_{1} } \right)$ denotes the right-hand rotation by ${\upgamma }_{i}$ with axis ${\mathbf{t}}_{{\mathbf{i}}}$, and ${\upgamma }_{i}$ is the fold angle along the crease with ${\mathbf{t}}_{{\mathbf{i}}}$ as its tangent. This leads to the following constraint on the angles ${\upgamma }_{i}$, which is necessary and sufficient for these folding angles to furnish a valid deformation:

4 $${\mathbf{R}}_{{{\mathbf{t}}_{1} }} \left( {{\upgamma }_{1} } \right) \cdots {\mathbf{R}}_{{{\mathbf{t}}_{6} }} \left( {{\upgamma }_{6} } \right) = {\mathbf{I}}.$$

For the waterbomb unit cell, we choose the tangent vectors ${\mathbf{t}}_{{\mathbf{i}}}$ to match the crease pattern as in Fig. 1a, with the vertical creases corresponding to ${\mathbf{t}}_{1,4}$ and the tangents ordered in a counterclockwise fashion. We then exploit the symmetry of the crease pattern to enforce a highly symmetric deformation, constraining the angles further as follows:

$${\upgamma }_{1} = {\upgamma }_{4} = \gamma , \;\;\;{\upgamma }_{2} = {\upgamma }_{3} = {\upgamma }_{5} = {\upgamma }_{6} = \omega .$$

With these constraints, in addition to the constraint in Eq. (4), we find that the folding angles ${\upgamma }_{i}$ are parametrized by a single parameter ${\upomega }$ during the folding motion; in particular, we find that

$$\cos \gamma = \frac{1 + \cos \omega }{{3 + \cos \omega }},\;\;\;\sin \gamma = \frac{ - 2\sqrt 2 \sin \omega }{{3 + \cos \omega }}.$$

Note, the parameterization above implies the relationship $\tan (\gamma /2) = \left( {1/\sqrt 2 } \right)\tan (\omega /2)$, which is the kinematic description provided elsewhere^21^ for symmetric waterbomb origami. By rescaling the tangent vectors appropriately, we obtain the parametrization for the vertices ${\mathbf{y}}_{0} , \ldots ,{\mathbf{y}}_{6}$ of the unit cell up to an overall rotation and translation. We provide an explicit parameterization of these vertices in the supplementary.

### Helical groups

The helical symmetry of the folded structure is encoded by a particular family of groups of isometries of ℝ^3^ known as the *helical groups*. Among these, those that are Abelian (i.e., those that satisfy the commutativity relation Eq. (2), the third condition) are generated by a pair of isometries ${\mathbf{g}}_{1} ,{\mathbf{g}}_{2} :{\mathbb{R}}^{3} \to {\mathbb{R}}^{3}$ parametrized by $\theta_{1} ,\theta_{1} \in \left( { - \pi ,\pi } \right]$, ${\uptau }_{1} ,{\uptau }_{2} \in {\mathbb{R}}$, ${\mathbf{e}} \in {\mathbb{S}}^{2}$, and ${\mathbf{z}} \in {\mathbb{R}}^{3}$ with ${\mathbf{e}} \cdot {\mathbf{z}} = 0$:

$${\mathbf{g}}_{{\mathbf{i}}} \left( {\mathbf{x}} \right) = {\mathbf{R}}_{{\mathbf{e}}} \left( {\theta_{i} } \right)\left( {{\mathbf{x}} - {\mathbf{z}}} \right) + \tau_{i} {\mathbf{e}} + {\mathbf{z}},\;\;\;i = 1,2,$$

where ${\mathbf{R}}_{{\mathbf{e}}} \left( {\theta_{1,2} } \right)$ are the 3 × 3 rotation matrices with axis ${\mathbf{e}}$ and angles $\theta_{1} ,\theta_{2}$, respectively. The group product is given by the composition of maps so that the helical group, which we denote by ${\mathcal{G}}$, is simply a subgroup of the Euclidean group on ${\mathbb{R}}^{3}$ generated by ${\mathbf{g}}_{1}$ and ${\mathbf{g}}_{2}$.

The application of these groups in our setting is as follows: Let us fix a folded origami unit cell ${\mathbf{y}}_{{\upomega }} \left( {\Omega } \right)$ defined as above with vertices ${\mathbf{y}}_{1} , \ldots ,{\mathbf{y}}_{6}$. For local compatibility of the structure (physically, this means that each unit cell will fit together with its neighbors to form a continuous structure, but the overall structure could exhibit unphysical self-intersection between cells that are far away from each other in the flat crease pattern), it is necessary and sufficient to satisfy the following four *local compatibility conditions*:

$${\mathbf{g}}_{1} \left( {{\mathbf{y}}_{2} } \right) = {\mathbf{y}}_{6} , \;\;\; {\mathbf{g}}_{1} \left( {{\mathbf{y}}_{3} } \right) = {\mathbf{y}}_{5} ,\;\;\;{\mathbf{g}}_{2} \left( {{\mathbf{y}}_{3} } \right) = {\mathbf{y}}_{1} ,\;\,\;{\mathbf{g}}_{2} \left( {{\mathbf{y}}_{4} } \right) = {\mathbf{y}}_{6} .$$

These equations can be viewed as constraints on the group parameters, and generically the problem of solving for the group parameters as described above in terms of the vertices ${\mathbf{y}}_{1} , \ldots ,{\mathbf{y}}_{6}$ can be fairly involved. However, due to the highly symmetric deformation of the unit cell that we enforce, we note that the parameter ${\mathbf{e}}$ is simply such that ${\mathbf{e}}\parallel {\mathbf{y}}_{2} - {\mathbf{y}}_{3}$, or equivalently ${\mathbf{e}}\parallel {\varvec{y}}_{5} - {\varvec{y}}_{6}$ or ${\mathbf{e}}\parallel {\mathbf{y}}_{1} - {\mathbf{y}}_{4}$. This identity allows us to recover the explicit formula (as described in the Supplementary) for the group parameters.

## Experimental

### Fabrication

The thin-films were sputtered using the Von Ardenne CS 730S magnetron cluster sputter system. The composition of the target used for sputtering was Ti_55.6_Ni_44.4_ at%. This was chosen to obtain a film composition of approximately Ti_50_Ni_50_ at% after accounting for elemental losses during sputtering. The samples were crystallized and shape set as cylinders in Rapid Thermal Annealing (RTA) at a temperature of 550 °C. This heat treatment was optimized to adjust the film composition to get the required transformation temperatures with martensite at room temperature.

### Characterization

The thin-films were manually folded using tweezers to their initial folded state. They were loaded in a silicon tube with a 2 mm diameter (catheter) and placed in a borosilicate glass tube of 6.3 mm diameter (vessel). For consequent expansion, the films were removed from the silicon tube. A self-made setup using temperature control (Eurotherm Controls) was used for the expansion experiments on heating.

### Force measurement

The force required to expand the stent (Design-01) was determined using a clinically available balloon catheter. The partially expanded stent in metastable state after heating to 150 °C was placed in glass tubes of 4.8 mm diameter. A balloon catheter (Invatec) with a diameter of 5 mm and a length of 20 mm was placed inside the implant. After which, pressurized Argon was introduced inside the balloon to inflate it. The balloon was inflated from a starting pressure of 1 bar (10^5^ N/mm^2^), and the stent was checked at the intervals of 0.5 bar. For the pressures of 1 bar and 1.5 bar, the implant did not recover its shape. At 2 bar, complete shape recovery was seen for the implant. The force of 27 N can be calculated as the dimension of the stent are known (diameter: 4.8 mm; length: 9 mm).

## Supplementary Information

Supplementary Information 1.

Supplementary Video 1.
