# Supplementary material for: Origami-inspired thin-film shape memory alloy devices
Source: Sci Rep. 2021 May 26;11:10988. doi: 10.1038/s41598-021-90217-3 (PMC8155017; doi:10.1038/s41598-021-90217-3)
Supplement: Supplementary file 1 — Supplementary Information 1. [file 41598_2021_90217_MOESM1_ESM.docx]

**Supplementary Information for**

Origami-inspired thin-film shape memory alloy devices.

Prasanth Velvaluri^1^, Arun Soor^2^, Paul Plucinsky^3^, Richard D. James ^4^, Rodrigo Lima de Miranda^5^, Eckhard Quandt*^1^

^1^Chair for Inorganic Functional Materials, Faculty of Engineering, Kiel University, Kiel, Germany.

^2^Mathematics Institute, University of Oxford, Oxford, UK.

^3^Aerospace and Mechanical Engineering, University of Southern California, Los Angeles, USA.

^4^Aerospace and Mechanical Engineering, University of Minnesota, Minneapolis, USA.

^5^Acquandas GmbH, Kiel, Germany.

**Corresponding author email:** [eq@tf.uni-kiel.de](mailto:eq@tf.uni-kiel.de)

We now present the formulas for waterbomb origami in a convenient and explicit form. Note, Note, these formulas are based on the systematic and rigorous characterization of the group theory method for helical groups provided in reference [15] in the main text.

**The symmetric kinematics of the waterbomb tube.** The symmetric waterbomb origami unit cells $\mathbf{y}_{\omega}\left( \Omega\right),$ defined in the main text, are composed of one interior vertex $\mathbf{y}_{0}$ and six vertices on the boundary $\mathbf{y}_{i}\boldsymbol{,}i=1,\ldots,6$. These vertices can be parameterized as

$$\mathbf{y}_{0}=\left( 0, 0, \frac{1}{4\sqrt{2}}\left( 1+\cos\left[ \omega\right] \right)\sqrt{3+\cos\left[ \omega\right]}\csc\left[ \frac{\omega}{2} \right] \right)^{T},$$

$$\mathbf{y}_{1}\boldsymbol{=}\left( 0, {\cos\left[ \frac{\omega}{2} \right]}^{2}, \frac{1}{4}\left( 3-\cos\left[ \omega\right] \right)\cot\left[ \frac{\omega}{2} \right]\sqrt{1+{\sec\left[ \frac{\omega}{2} \right]}^{2}} \right)^{T},$$

$$\mathbf{y}_{2}= \left( -\frac{2\cos\left[ \frac{\omega}{2} \right]}{\sqrt{3+\cos\left[ \omega\right]}}, 1, \frac{\left( 15+\cos\left[ 2\omega\right] \right)\csc\left[ \frac{\omega}{2} \right]}{8\sqrt{2}\sqrt{3+\cos\left[ \omega\right]}} \right)^{T},$$

$$\mathbf{y}_{3}=\left( -\frac{2\cos\left[ \frac{\omega}{2} \right]}{\sqrt{3+\cos\left[ \omega\right]}}, -1, \frac{\left( 15+\cos\left[ 2\omega\right] \right)\csc\left[ \frac{\omega}{2} \right]}{8\sqrt{2}\sqrt{3+\cos\left[ \omega\right]}} \right)^{T},$$

$$\mathbf{y}_{4}=\left( 0, -{\cos\left[ \frac{\omega}{2} \right]}^{2}, \frac{1}{4}\left( 3-\cos\left[ \omega\right] \right)\cot\left[ \frac{\omega}{2} \right]\sqrt{1+{\sec\left[ \frac{\omega}{2} \right]}^{2}} \right)^{T},$$

$$\mathbf{y}_{5}= \left( \frac{2\cos\left[ \frac{\omega}{2} \right]}{\sqrt{3+\cos\left[ \omega\right]}}, -1, \frac{\left( 15+\cos\left[ 2\omega\right] \right)\csc\left[ \frac{\omega}{2} \right]}{8\sqrt{2}\sqrt{3+\cos\left[ \omega\right]}} \right)^{T},$$

$$\mathbf{y}_{6}=\left( \frac{2\cos\left[ \frac{\omega}{2} \right]}{\sqrt{3+\cos\left[ \omega\right]}}, 1, \frac{\left( 15+\cos\left[ 2\omega\right] \right)\csc\left[ \frac{\omega}{2} \right]}{8\sqrt{2}\sqrt{3+\cos\left[ \omega\right]}} \right)^{T}$$

in the standard basis, where the superscript *T* denotes the transpose operation. Note, the vertices here depend implicitly on the folding parameter $0\leq\omega\leq\pi$. When $\omega=0,$ we obtain the flat cell $\Omega$ shown in Fig. 1(a). Evolving the folding parameter from $\omega=0$ to $\pi$yields the vertices of the folded cells exactly as described in Fig. 1(b) with $\mathbf{e=}\left( 0,1,0 \right)^{T}.$

For each $\omega$ under the parameterization above, there is a unique set of isometries $\mathbf{g}_{1,2}$ that obey the rules in Eq. (1-2). The parameterization$\mathbf{g}_{1,2}\boldsymbol{=}\mathbf{g}_{1,2}\left( \omega\right)$ is also continuous in $\omega$. Specifically, the isometries are given by rotations and translations

$\mathbf{R}_{\boldsymbol{i}}\boldsymbol{=}\left( \begin{matrix} cos[\theta_{i}] & 0 & sin[\theta_{i}] \\ 0 & 1 & 0 \\ -sin[\theta_{i}] & 0 & cos[\theta_{i}] \end{matrix} \right)\boldsymbol{,}\mathbf{b}_{i}\boldsymbol{=}\left( \begin{matrix} 0 \\ \tau_{i} \\ 0 \end{matrix} \right), i=1,2,$

for which the twist angles $\theta_{i}$ and axial displacements $\tau_{i}$ satisfy

$$\theta_{1}=\mathrm{sign}\left[ \mathbf{e}\cdot\left( \mathbf{y}_{2}\boldsymbol{\times}\mathbf{y}_{6} \right) \right]\arccos\left[ \frac{\mathbf{P}_{\mathbf{e}}\mathbf{y}_{6}\cdot\mathbf{P}_{\mathbf{e}}\mathbf{y}_{2}}{\left| \mathbf{P}_{\mathbf{e}}\mathbf{y}_{6} \right|\left| \mathbf{P}_{\mathbf{e}}\mathbf{y}_{2} \right|} \right],$$

$$\theta_{2}=\mathrm{sign}\left[ \mathbf{e}\cdot\left( \mathbf{y}_{3}\boldsymbol{\times}\mathbf{y}_{1} \right) \right]\arccos\left[ \frac{\mathbf{P}_{\mathbf{e}}\mathbf{y}_{1}\cdot\mathbf{P}_{\mathbf{e}}\mathbf{y}_{3}}{\left| \mathbf{P}_{\mathbf{e}}\mathbf{y}_{1} \right|\left| \mathbf{P}_{\mathbf{e}}\mathbf{y}_{3} \right|} \right],$$

$$\tau_{1}=\mathbf{e}\cdot\left( \mathbf{y}_{6}-\mathbf{y}_{2} \right),$$

$$\tau_{2}=\mathbf{e}\cdot\left( \mathbf{y}_{1}-\mathbf{y}_{3} \right),$$

with $\mathbf{P}_{\mathbf{e}}\boldsymbol{=}\mathbf{Id-e}\otimes\mathbf{e}$ and $\boldsymbol{|}\cdot|$ denoting the Euclidean distance. Since these group parameters depend on the vertices $\mathbf{y}_{i}$, they depend implicitly on the folding parameter $\omega.$

The waterbomb origami structures folded from the crease pattern in Fig. 1(a) under this parameterization are

$$\left\{ \mathbf{g}_{1}^{p}\mathbf{g}_{2}^{q}\left( \mathbf{y}_{\omega}\left( \Omega\right) \right):\left( p,q \right)\in\{\left( 1,1 \right), \ldots,\left( 1,6 \right),\left( 2,1 \right),\ldots,\left( 2,6 \right),\left( 3,0 \right),\ldots,(3,5)\} \right\}$$

for $0\leq\omega\leq\pi,$ where $\mathbf{g}_{1}^{p}\mathbf{g}_{2}^{q}\left( \mathbf{x} \right)\boldsymbol{=}\mathbf{R}_{1}^{p}\mathbf{R}_{2}^{q}\mathbf{x+}\left( p\tau_{1}+q\tau_{2} \right)\mathbf{e.}$ The fully crimped state is given by $\omega=\pi$. By evolving $\omega$ from $\pi$ to $\approx0.63\pi$, we unroll the waterbomb origami from the crimped state to the most compact tube shown in Fig. 5. Evolving the folding angle further gives two regimes: (i) the regime that takes $\omega$ from $\approx0.63\pi$ to $\approx0.37\pi,$ where the ends of the tube overlap in an unphysical way until reaching the middle tube shown in Fig. 5; (ii) the regime that takes $\omega$ from $\approx0.37\pi$ to 0, where the middle tube Fig. 5 unrolls by flattening out into the flat crease pattern shown in 1(a).

**Transition to vertical crease folding.** To obtain the large radius tube in Fig. 1(d) by a continuous family of ideal origami deformations from the crimped state, one must first flatten out the pattern, as described above. From the flat crease pattern, the final tube arises by folding solely along the vertical creases shown in Fig. 1(a). For completeness, the natural symmetric folding process to obtain this tube can be parameterized by unit cells $\mathbf{y}_{\gamma}\left( \Omega\right)$ with vertices

$$\mathbf{y}_{0}=\left( 0, 0, \frac{1}{2}\csc[\frac{\gamma}{2}] \right)^{T},$$

$$\mathbf{y}_{1}\boldsymbol{=}\left( 0, 1, \frac{1}{2}\csc[\frac{\gamma}{2}] \right)^{T},$$

$$\mathbf{y}_{2}= \left( -\cos\left[ \frac{\gamma}{2} \right], 1, \frac{1}{2}\cos[\gamma]\csc[\frac{\gamma}{2}] \right)^{T},$$

$$\mathbf{y}_{3}=\left( -\cos\left[ \frac{\gamma}{2} \right], -1, \frac{1}{2}\cos[\gamma]\csc[\frac{\gamma}{2}] \right)^{T},$$

$$\mathbf{y}_{4}=\left( 0, -1, \frac{1}{2}\csc[\frac{\gamma}{2}] \right)^{T},$$

$$\mathbf{y}_{5}= \left( \cos\left[ \frac{\gamma}{2} \right], -1, \frac{1}{2}\cos[\gamma]\csc[\frac{\gamma}{2}] \right)^{T},$$

$$\mathbf{y}_{6}=\left( \cos\left[ \frac{\gamma}{2} \right], 1, \frac{1}{2}\cos[\gamma]\mathrm{Csc}[\frac{\gamma}{2}] \right)^{T}.$$

The isometries $\mathbf{g}_{1,2}$ are then defined by exactly the same formulas above, only under this new parameterization for the vertices. Finally, the overall origami is given by

$$\left\{ \mathbf{g}_{1}^{p}\mathbf{g}_{2}^{q}\left( \mathbf{y}_{\gamma}\left( \Omega\right) \right):\left( p,q \right)\in\{\left( 1,1 \right), \ldots,\left( 1,6 \right),\left( 2,1 \right),\ldots,\left( 2,6 \right),\left( 3,0 \right),\ldots,(3,5)\} \right\}$$

for $0\leq\gamma\leq\pi/5.$ Note, evolving the folding parameter $\gamma$ from 0 to $\pi/5$ takes the origami continuously from the flat crease pattern in 1(a) to the large radius tube shown in 1(d).

**Design of the considered Origami implant:**

Table S1. Calculated porosity of the designs 01-03.

|  | Open area  (mm^2^) | $Porosity (\%)= \frac{Open}{Total}*100$ |
| --- | --- | --- |
| Design-01 | 0.0424 | 0.47 |
| Design-02 | 2.076 | 23.06 |
| Design-03 | 3.0437 | 33.81 |
